# Umbilical Cord Blood Endothelial Progenitor Cells Modulate Neurovascular Unit Damage in a Neonatal Rat Model of Hypoxia Ischemia

**DOI:** 10.1007/s12015-025-10939-z

**Published:** 2025-07-31

**Authors:** Tayla R. Penny, Yen Pham, Amy E. Sutherland, Graham Jenkin, Tamara Yawno, Suzanne L. Miller, Courtney A. McDonald

**Affiliations:** 1 The Ritchie Centre, Hudson Institute of Medical Research, Clayton, Victoria Australia; 2 Department of Obstetrics and Gynaecology, Monash University, Clayton, Victoria Australia; 3 Department of Paediatrics, Monash University, Clayton, Victoria Australia

**Keywords:** Perinatal brain injury, Stem cells, Cerebral palsy, Cell therapy, Neuroprotection

## Abstract

**Introduction:**

Neonatal hypoxia ischemia (HI) causes injury to the blood brain barrier (BBB), which in turn is associated with widespread cell loss. Cell therapies are currently being investigated for use in perinatal neuroprotection and umbilical cord blood (UCB) endothelial progenitor cells (EPCs; CD133+) have been previously shown to reduce gross neuropathology associated with HI; however, no study has investigated the effect of EPC treatment on the vulnerable BBB. Therefore, in this study, we investigated the effect of EPC treatment on BBB integrity in a neonatal rat model of HI.

**Methods:**

HI brain injury was induced in postnatal day (PND) 7 rat pups via permanent ligation of the left carotid artery, followed by a 180-minute hypoxic challenge at 8% O_2_. At 24 h post-insult, 2 × 10^5^ EPCs were administered via intraperitoneal injection. At 7 days post-HI, brains and choroid plexuses were collected for immunohistochemistry or molecular analyses.

**Results:**

Neonatal HI resulted in neuropathology, with a significant increase in left hemisphere tissue loss. This was associated with microglial activation, astrogliosis and increased expression of Aquaporin 4 in the somatosensory cortex, and EPC administration significantly reduced these outcomes. Assessment of the BBB revealed GLUT1 and claudin-5 expression were significantly increased in HI animals compared to sham, and treatment with EPCs reduced these to sham control levels.

**Conclusion:**

EPCs are a promising treatment option for neonatal HI as they modulate BBB changes. However, further studies are needed to understand early effects of EPC treatment and how EPC therapy modulates BBB dysfunction post HI brain injury.

**Graphical Abstract:**

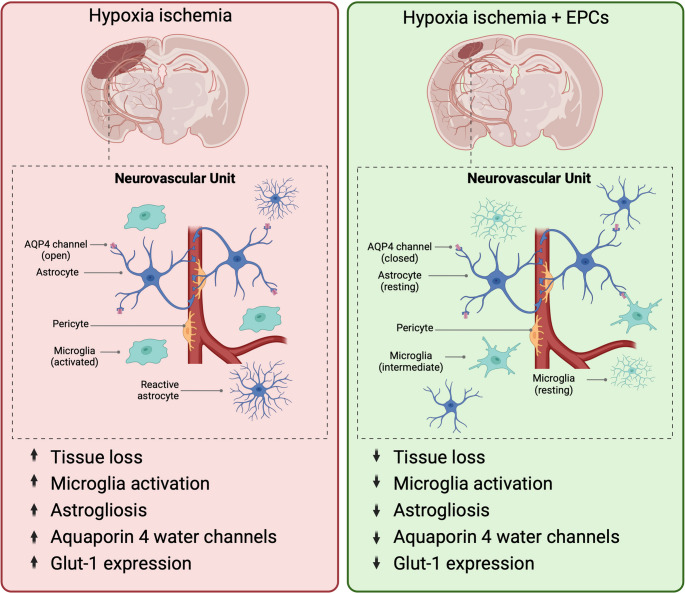

**Supplementary Information:**

The online version contains supplementary material available at 10.1007/s12015-025-10939-z.

## Introduction

Neonatal hypoxic ischemic (HI) brain injury is caused by disruption of blood and oxygen supply during gestation or birth and can lead to lifelong neurological deficits, including cerebral palsy [1]. There are a multitude of neurological consequences of HI, one of which is injury to the blood brain barrier (BBB), a structure comprised of endothelial cells, pericytes, a basement membrane and astrocytic end feet [2]. Further to this, the BBB functions as part of the neurovascular unit (NVU), which more broadly includes the interactions between neurons, microglia and astrocytes. These cells work with the BBB to maintain cerebral homeostasis and tightly regulate neuroimmune responses and vascular function [3]. In the setting of HI brain injury, the NVU is susceptible to injury, including damage to the BBB and dysregulation of endothelial cells. Specifically, HI results in increased BBB permeability, predominantly in the first 48 h in rodents [4–6] and sheep [7–9], and in turn results in infiltration of red blood cells and peripheral immune cells into the brain tissue. Cellular components of the NVU, including astrocytes, neurons and microglia, are also affected by neonatal HI, resulting in neuron loss, microglial activation and astrogliosis [10].

Currently, the only clinically available therapy for HI brain injury is therapeutic hypothermia, however this is not always feasible, as it can only be applied to term born infants, and ~ 40% of cooled infants still go on to experience significant morbidity and mortality [11]. Further, the effect of therapeutic hypothermia on the injured BBB is not well characterised thus, it is unclear if hypothermia is protective of the brain vasculature [12]. Therefore, novel treatment strategies are being pursued for neonatal HI that can be applied as a standalone therapy or an adjuvant therapy alongside therapeutic hypothermia to increase therapeutic efficacy and overcome restrictions currently associated with hypothermia.

Umbilical cord blood (UCB) cells have been studied as a potential treatment option for a variety of neurological conditions, particularly in the neonate, including injury resulting from HI [13]. UCB contains a heterogeneous population of stem and progenitor cells, including haematopoietic stem cells (HSCs; CD34+), T regulatory cells (Tregs; CD4 + CD25+), monocytes (CD14+) and endothelial progenitor cells (EPCs; CD133+) [14]. We have previously investigated the efficacy of whole UCB mononuclear cells (MNCs) compared with individual UCB derived cell types in a neonatal rodent model of HI and we concluded that EPCs were able to modulate neuroinflammation, reduce brain injury and modulate behavioural deficits to a similar degree when compared to the whole UCB mononuclear fraction of cells [15]. Another study by Kidani et al. demonstrated that UCB CD133 + cells migrated to the ipsilateral hemisphere in a mouse model of neonatal HI and reduced tissue loss and cystic lesion size and improved long-term motor deficits [16]. Other preclinical studies demonstrated that EPCs reduced astrogliosis and brain infarct size and down regulated the expression of pro-inflammatory genes known for driving injury, tumour necrosis factor-α (TNFα), interleukin (IL)−1β and IL-6 [17]. They also demonstrated pro-angiogenic effects, with EPC treated animals exhibiting increased vessel density [18], increased capillary density and upregulation of vascular endothelial growth factor (VEGF) [19].

Whilst we and others have shown improvements in neuropathology following EPC administration, the effects of EPC treatment on the BBB in neonatal HI have not been investigated. Given that EPCs have shown to have neuroprotective properties, as discussed above, it is likely that EPCs provide protective benefits on the cellular and subcellular components of the BBB. As such, in this present study, we utilised a well-described rat model of neonatal HI brain injury in postnatal day (PND) 7 rat pups [15] to assess the therapeutic potential of UCB derived CD133 + EPCs administered intraperitoneally 24 h post-HI. Due to the anti-inflammatory and angiogenic nature of these cells, we hypothesised that these EPCs would modulate the effects of neonatal HI, specifically by reducing injury to the NVU by modulating BBB dysfunction.

## Methods

### Ethics Approval

All experiments in this project were performed with human and animal ethics approval from Monash Health Human Ethics Committee (12387B) and Monash Medical Centre Animal Ethics Committee A (MMCA/2015/42), respectively. All experiments were performed in accordance with the ARRIVE guidelines 2.0.

### Cell Preparation

#### UCB Mononuclear Cell Isolation

Human UCB samples were obtained from women with uncomplicated pregnancies undergoing elective caesarean section at term (> 37 weeks gestation) who gave written, informed consent for the collection of their UCB. After clamping of the cord and delivery of the placenta, UCB was collected from the umbilical vein using blood collection bags containing anticoagulant (Macopharma, #MSC1201DU, Tourcoing, France). On average, ~ 100 ml of UCB was collected and stored at room temperature < 48 h until processing and isolation. UCB mononuclear cells were isolated as previously described [15, 20]. Briefly, UCB samples were centrifuged, and the buffy coat containing the mononuclear cells was collected. Red blood cell lysis was performed to remove contaminating erythrocytes. The mononuclear cells were counted using a haemocytometer and used for magnetic bead separation of CD133 + cells (hereafter referred to as EPCs).

#### Magnetic Activated Cell Sorting (MACS)

EPCs were isolated using CD133 + MACS beads (Miltenyi Biotec, #130-097-049, Germany). All procedures were performed according to the manufacturer’s instructions. Following isolation, purity was assessed via flow cytometry and all isolations were confirmed to have > 80% purity. Following isolation, cells were cryopreserved for later use at a density of 1–2 × 10^6^ cells/ml in 40% complete media (DMEM/F12, 16.5% FBS, 1% antibiotics), 50% fetal bovine serum (FBS; #SFBS, Bovogen, New Zealand), and 10% dimethyl sulfoxide (DMSO; #D2650, Sigma-Aldrich, MO, USA).

### Animals

Time-mated pregnant Sprague-Dawley (SD) rat dams were sourced from Monash Animal Research Platform and transported to Monash Medical Centre Animal Facility one week prior to birth. They were housed in individual boxes in standard housing conditions in rooms with a 12 h light/dark cycle. The dams were allowed to birth naturally and were only disturbed to count the pups on PND 2–3. Litter size was standardised to 8–12 pups per litter to ensure equal care of all pups. A total of 36 rat pups (from 7 dams) were used in this study. Rat pups were randomly assigned to experimental groups, based on surgery times, to control for any neuroprotective effects associated with prolonged isoflurane exposure during anaesthesia, and animals from each litter were distributed amongst groups to control for litter variation. 24 animals were exposed to HI (HI only, *n* = 11; HI + EPC, *n* = 13), and 12 animals received sham surgery with no hypoxia exposure (Sham). A power calculation was performed to determine optimal group size (alpha = 0.05, power = 0.8, minimum 4 pups per group). The sex of the pups was not considered when assigning groups, and further we are underpowered in this study to detect any sex differences.

#### Animal Surgery

As previously described [15], we used the Rice-Vannucci model to induce term neonatal HI, at PND 7. Pups were maintained on a heating mat (37 °C) throughout the surgery. Under anaesthesia (2% isoflurane, Abbott, Australia), a midline incision was made in the neck of the pups, and the left common carotid artery was permanently occluded using a cautery device. The incision was closed, and pups returned to their mother for at least 1 h to recover. The average time under anaesthesia was 8 min per pup. Subsequently, pups were placed into a hypoxic chamber for 180 min (BioSpherix, Lacona, NY; 8% oxygen, temperature controlled at 36 °C). Control pups underwent sham surgery and were allowed to recover for 1 h with their mother, then were removed for the same duration as the HI animals and kept in room air on a heating pad at 37 °C. Pup weights and general wellbeing were monitored daily from PND 7 until PND 14. There was no mortality observed in this study.

#### Cell Treatment

24 h post HI injury (PND 8), EPCs were administered by intraperitoneal injection. Cells were thawed and pooled from a minimum of three donors to reduce the potential for donor variation. Cell viability prior to administration was examined and was > 80% for all animals. Pups received 200,000 EPCs in 200 µl phosphate-buffered saline (PBS) using a 30-gauge insulin syringe. HI injury control pups received 200 µl PBS alone.

#### Postmortem and Tissue Processing

On PND 14, animals were culled using an overdose of pentobarbitone sodium (0.1 mg/g, Valabarb, Australia). Brains were collected and weighed before being immersion fixed in 10% formalin (48 h at room temperature; Amber Scientific) for histology (sham, *n* = 8; HI, *n* = 7; HI + EPC, *n* = 9), or choroid plexuses were dissected from brains and snap frozen in liquid nitrogen for RNA extraction (*n* = 4/group). Separate animals were used for histological and molecular analyses.

### Immunohistochemical Assessment

Paraffin-embedded coronal brain sections were cut at 6 μm and sections included in analysis were approximately − 3.5 to −4.5 mm from bregma. Sections were analysed by immunohistochemistry or immunofluorescence using the following primary antibodies, incubated overnight at 4 °C: ionised calcium-binding adapter molecule 1 (Rabbit anti-Iba-1; 1:1000, Wako Pure Chemical Industries, Ltd. Osaka, Japan), laminin (Rabbit anti-laminin; 1:200, NB300-144, Novus Biologicals, CO, USA), glucose transporter 1 (Rabbit anti-GLUT1; 1:200, #AB14683, Abcam, Cambridge, UK), Claudin-5 (Mouse anti-Claudin-5; 1:100, #35-2500, ThermoFisher Scientific, MA, USA), glial fibrillary acidic protein (Mouse anti-GFAP;1:500, #G3893, Sigma-Aldrich, MO, USA), aquaporin-4 (Rabbit anti-AQP4; 1:200, #AB2218, Sigma-Aldich, MO, USA) and rat albumin (Sheep anti-rat albumin; 1:2000, #A110-134 A, ThermoFisher Scientific, MA, USA). All sections were exposed to a secondary antibody for 1 h at room temperature (1:200; biotinylated goat anti-rabbit, goat anti-mouse, rabbit anti-sheep, Vector Laboratories, Burlingame, CA, USA, goat anti-mouse Alexa Fluor 488 and goat anti-rabbit Alexa Fluor 594, #A11005 and #A11008, ThermoFisher Scientific, MA USA). For non-fluorescent chromogenic immunohistochemistry, staining was visualised using 3,3-diaminobenzidine (MP Biomedicals, Santa Ana, CA, USA). Slides were imaged at 400× magnification using bright field microscopy on an Olympus BX-41 53 microscope (OlympusEvident Scientific/Olympus, Tokyo, Japan). The somatosensory cortex of coronal brain sections was analysed in duplicates, with three fields of view analysed per section (total 6 per slide; fields of view = 0.139mm^2^). Cell counts were performed using QuPath imaging software (University of Edinburgh, Scotland, UK, version 0.3.0), and densitometry was performed using a macro in Image J (NIH, Bethesda, USA). Vessel density was determined by counting vessels in laminin-stained sections. Quantification of microglia cell types was achieved by classing the microglia as either resting (ramified, with branching projections protruding from the cell body), intermediate (bushy, a singular dendritic projection from the cell body or short/retracting processes) or activated (ameboid, with a round distinct cell body with no dendritic projections) as previously described [21]. All assessments were conducted on coded slides and images, with the examiner (TRP) blinded to the experimental groups.

#### Gross Brain Morphology

Gross brain morphology and tissue area were assessed with cresyl violet and acid fuchsin stain (Grale Scientific Pty Ltd., Victoria, Australia). For each animal, duplicate slides were assessed, and data was averaged across groups. Images were acquired by Aperio digital scanning (Olympus VS120 slide scanner, Leica Biosystems, Germany), and the area of the left (injured) hemisphere and the total brain area was measured using Qupath. To determine the tissue loss in the injured hemisphere, the area of the left hemisphere was divided by the area of the whole brain to obtain the percentage of the left hemisphere.

### mRNA Expression

The choroid plexus was dissected and snap frozen, and RNA was extracted for quantitative real-time PCR. Snap-frozen tissue was homogenised, and total RNA was isolated (Purelink RNA mini kit, Ambion, Life Technologies) and reverse-transcribed into cDNA (SuperScript III reverse transcriptase, Invitrogen; Life Technologies). Relative mRNA expression was measured by quantitative realtime (RT) PCR using Applied Biosystems 7900HT Fast Real-Time PCR system. The expression of all genes was normalized to the 18 S rRNA for each sample using the cycle threshold (ΔCT) method of analysis and was expressed relative to the sham control group. See supplementary Table 1 for RT-PCR primer sequences.

### Statistical Analysis

Results are expressed as the mean ± standard error of the mean (SEM). Statistical analysis was performed using Prism 10 (GraphPad Software). Significant outliers were removed (using Robust Outlier elimination (ROUT) with Q = 1%), and data were analysed using a one-way ANOVA with Tukey’s post-hoc analysis. A value of *P* < 0.05 was considered statistically significant.

## Results

### The Effect of Neonatal HI and EPC Administration on Gross Neuropathology

**Fig. 1:**
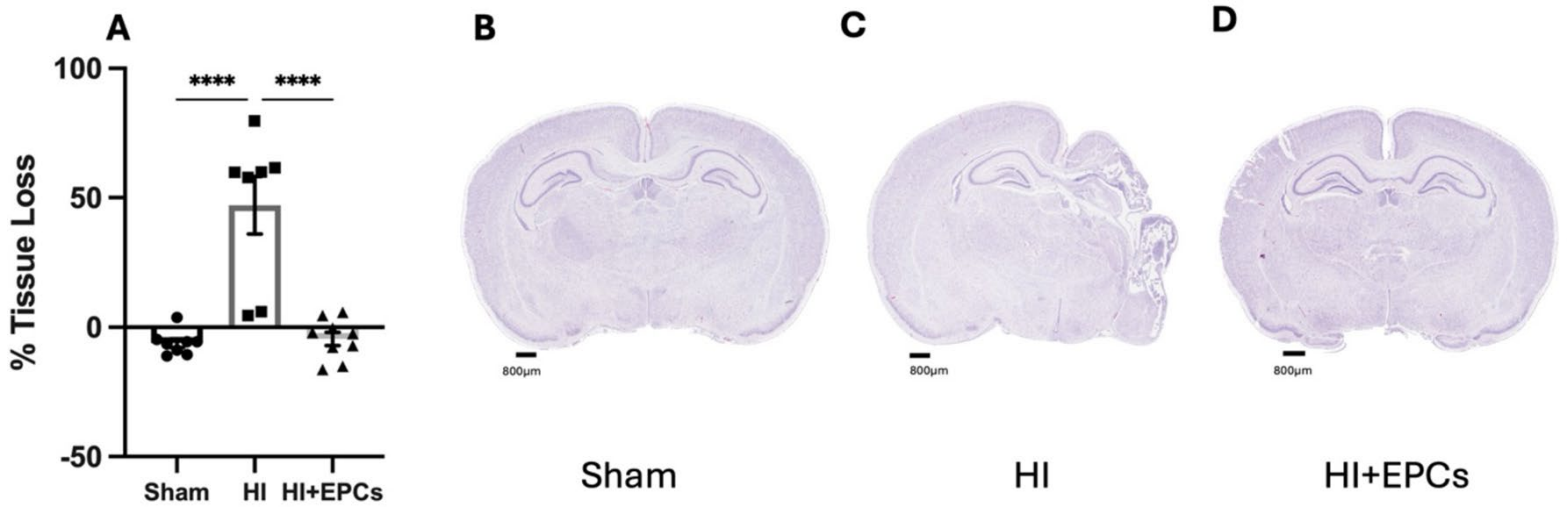
EPCs prevented ipsilateral tissue loss when administered 24 h after HI. (**A**) Cresyl violet- Acid fuchsin (CV/AF) staining was used to quantify ipsilateral hemisphere area tissue loss relative to total brain area. Sham, *n* = 8; HI, *n* = 7; HI + EPC, *n* = 9. ****P < 0.0001. All data are presented as mean ± SEM. Data were analysed using a one-way ANOVA with Tukey’s post-hoc analysis. Scale bar = 800 μm Representative CV/AF images of (**B**) sham, **C** HI and (**D**) EPC treated animals

We first confirmed that brain injury was evident 7 days post-HI by measuring ipsilateral hemisphere tissue loss. The HI group had significant severe tissue loss compared to the sham group (*P* < 0.0001; Fig. 1a-c). Treatment with EPCs significantly reduced tissue loss when compared to the HI group (*P* < 0.0001; Fig. 1a, d) and tissue loss in EPC treated animals was not different from sham animals.

### The Effect of EPC Administration on EPC Activation and Morphology

**Fig. 2:**
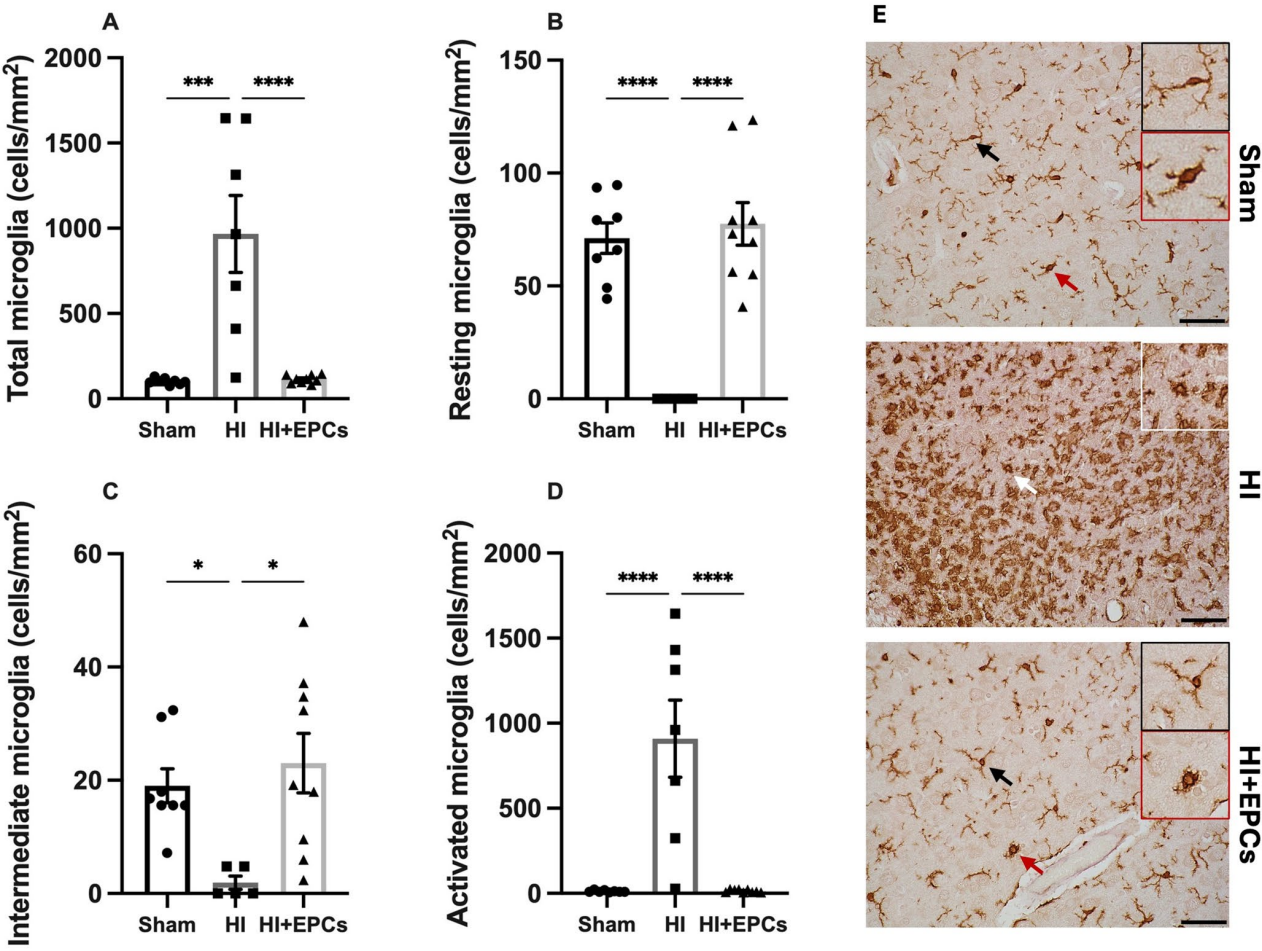
EPCs modulate neuroinflammation following neonatal HI. Immunohistochemistry was used to detect Iba1 + microglia. **A** Total Iba1 + microglia were quantified in the somatosensory cortex. Microglia were then further characterised as (**B**) resting with a ramified phenotype, (**C)** intermediate with a bushy phenotype, and (**D**) activated with an ameboid phenotype. Sham, *n* = 8; HI, *n* = 7; HI + EPC, *n* = 9. *P < 0.05, ***P < 0.001, ****P < 0.0001. All data are presented as mean ± SEM. Data were analysed using a one-way ANOVA with Tukey’s post-hoc analysis. **E** Representative images of Iba1 staining in sham, HI and EPC treated animals. Scale bar = 50 μm

The total number of microglia was significantly higher in the cortex of HI animals compared to sham (*P* = 0.0001), and EPC treatment significantly ameliorated this response (*P* < 0.0001; Fig. 2a, e). Microglia morphology was characterised in the somatosensory cortex to determine microglial activation following HI and EPC administration. Microglia were classified as either resting, intermediate or activated, as previously described [21]. Our results show that HI animals had significantly fewer resting and intermediate microglia compared to sham (*P* < 0.0001, *P* = 0.044, respectively) and EPC treated groups (*P* < 0.0001, *P* = 0.010, respectively; Fig. 2b, c). Conversely, the HI group displayed significantly more activated microglia compared to the sham (*P* < 0.0001) and EPC treated groups (*P* < 0.0001), demonstrating a neuroinflammatory state following HI which was significantly modulated by EPC treatment when given 24 h post HI (Fig. 2d).

### The Effect of EPC Administration on Astrogliosis and Aquaporin Expression

**Fig. 3:**
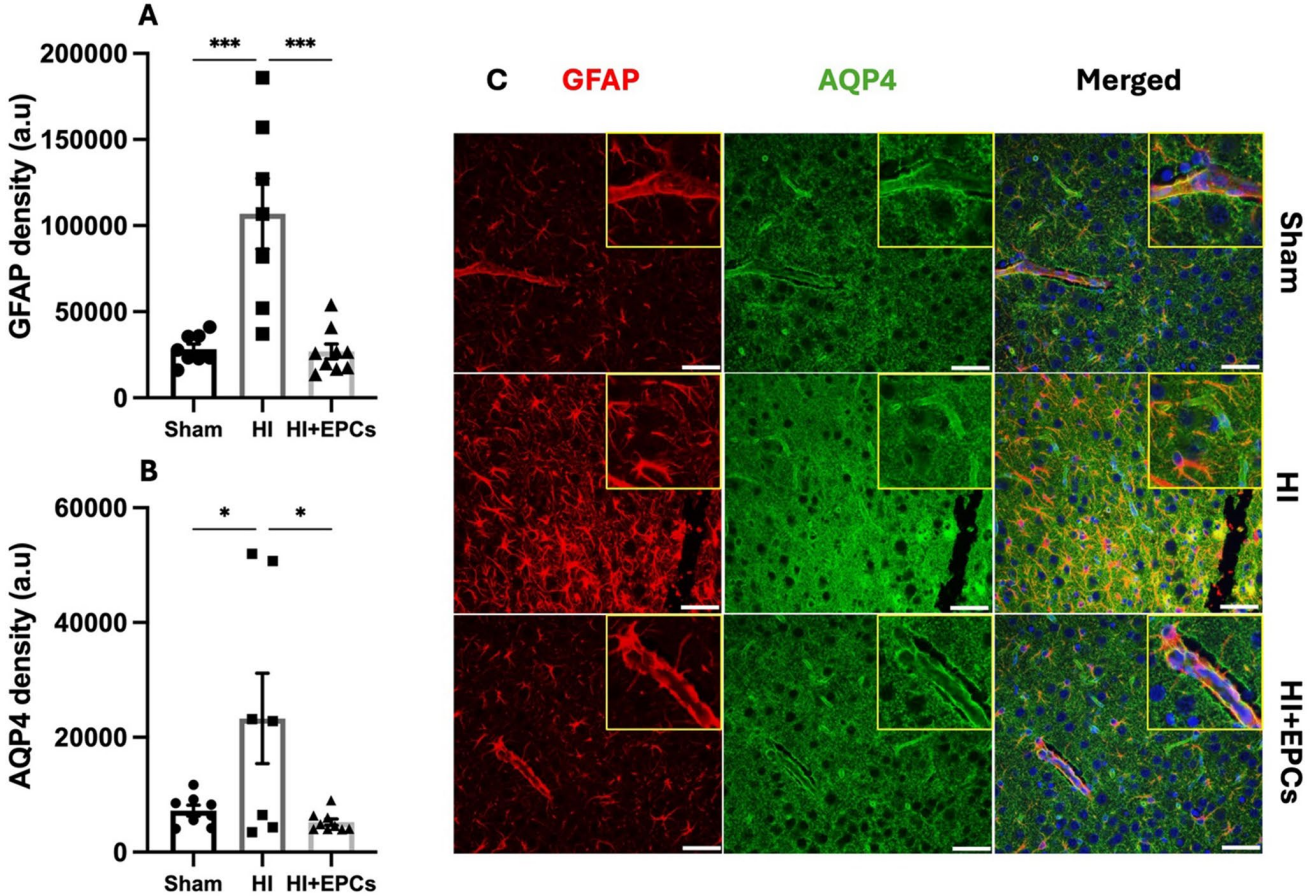
HI causes astrogliosis and increased water channel expression, both of which are reduced by EPC treatment. Double-label immunofluorescence was performed to assess (**A**) astrocyte density using glial fibrillary acidic protein (GFAP) staining and (**B**) aquaporin-4 (AQP4) density, an astrocyte specific water channel. Sham, *n* = 8; HI, *n* = 7; HI + EPC, *n* = 9. *P < 0.05, ***P < 0.001. All data are presented as mean ± SEM. Data were analysed using a one-way ANOVA with Tukey’s post-hoc analysis. Density is expressed as arbitrary units (a.u) and is measured per field of view (0.139mm^2^).'(**C)** Representative images of GFAP (red), AQP4 (green) and Hoescht (blue) staining in sham, HI and EPC treated animals. Scale bar = 50 μm

Astrocytes are known to contribute to neuroinflammation and exacerbation of brain injury. Here, we used GFAP immunofluorescence to detect astrocytes in the brain. There was a significant increase in GFAP expression in the HI group compared to the sham group (*P* = 0.0002; Fig. 3a). EPC treatment at 24 h post HI significantly ameliorated the increase in astrocyte activation compared to HI alone (*P* = 0.0001). We also measured the expression of AQP4 in the brain. AQP4 is a hydrophobic transmembrane protein that is abundantly expressed on astrocytes and plays a considerable role in oedema formation. In this study, there was a significant increase in AQP4 expression in the HI group compared to sham (*P* = 0.031; Fig. 3b). This was significantly modulated by EPC administration, resulting in a significant decrease in AQP4 expression in the EPC group compared to HI alone (*P* = 0.012). Additionally, there was a significant positive correlation between AQP4 and GFAP expression (*P* < 0.0001, R^2^ = 0.65; Fig. 3c, Supplementary Fig. 1).

### The Effect of Neonatal HI and EPC Administration on Markers of BBB Integrity

**Fig. 4:**
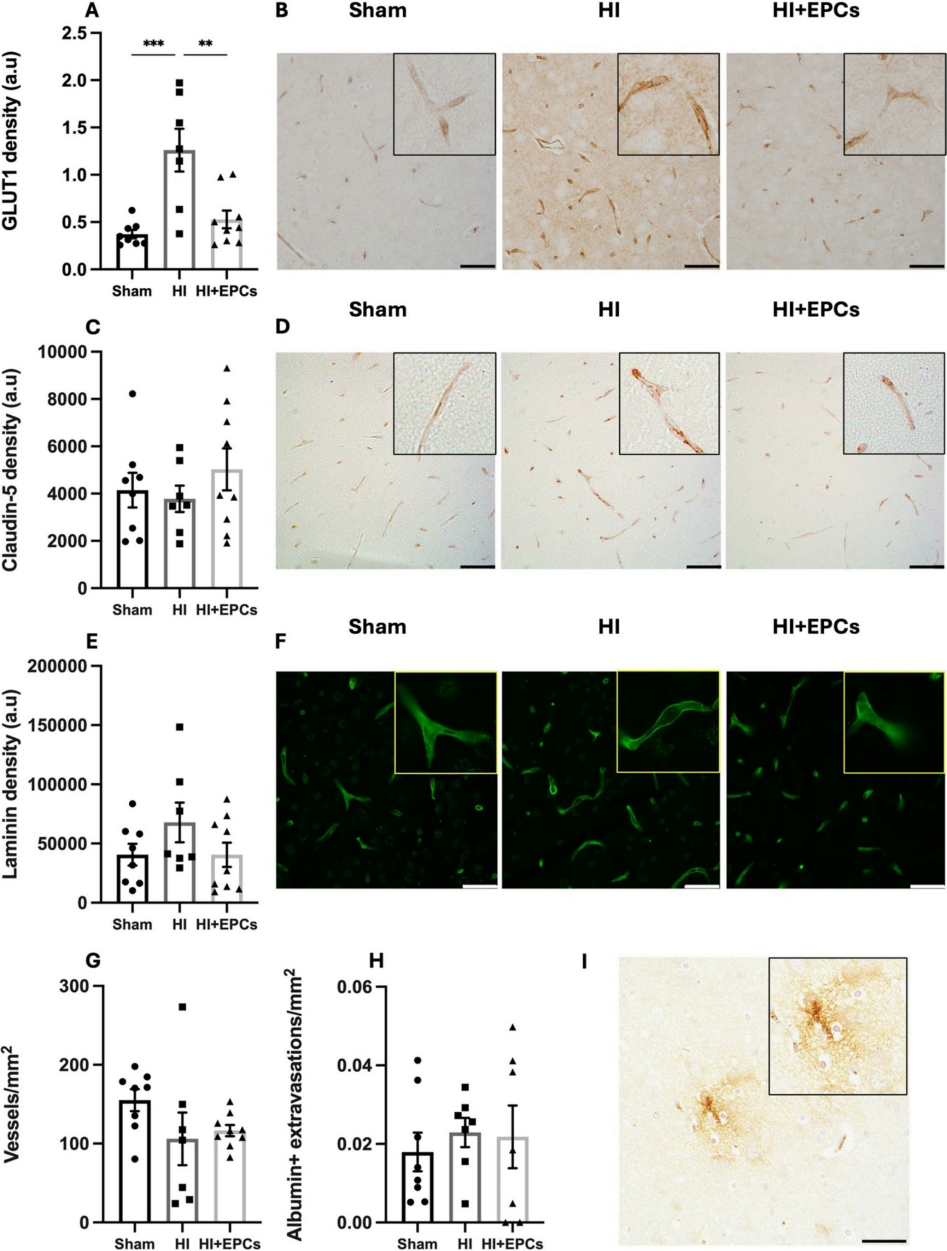
Exposure to HI resulted in mild BBB changes 7 days post injury. Components of the BBB were analysed in the somatosensory cortex using immunohistochemistry/immunofluorescence. Specifically, density of (**A**,** B**) the glucose transporterGLUT1, (**C**,** D)** claudin-5, a key tight junction protein found between endothelial cells and (**E**,** F**) laminin, a major component of the basal lamina. (**G)** The number of vessels in laminin-stained sections was also quantified, and (**H**,** I**) vessel leakiness was measured through albumin extravasation in the ipsilateral hemisphere. Density is expressed as arbitrary units (a.u) and is measured per field of view (0.139mm^2^). Sham, *n* = 8; HI, *n* = 7; HI + EPC, *n* = 9. **P < 0.01, ***P < 0.001. All data are presented as mean ± SEM. Data were analysed using a one-way ANOVA with Tukey’s post-hoc analysis. Scale bar = 50 μm

A potential neuroprotective effect of EPCs is via actions on cells of the NVU [22]. GLUT1 is a transporter that facilitates the movement of glucose across the cell membrane and is highly expressed in vascular endothelial cells of the BBB. We observed a significant increase in GLUT1 expression in the cortex following HI compared to sham (*P* = 0.0004; Fig. 4a, b) and EPC treatment significantly reduced GLUT1 expression compared to HI (*P* = 0.002). The EPC treated group was not different to the sham (Fig. 4a, b).

Claudin-5 is a key tight junction protein crucial for endothelial cell-cell adhesion in the neurovasculature. There was no difference in claudin-5 protein expression between any group (Fig. 4c, d). Laminins are extracellular matrix proteins and a major constituent of the basal lamina component of the NVU [23]. There was no significant difference in laminin expression between any treatment group (Fig. 4e, f).

We quantified the number of blood vessels using laminin-stained sections and there was no difference in vessel number between groups (Fig. 4g). We further measured vessel leakiness by quantifying the presence of albumin extravasations around vessels in the ipsilateral hemisphere. There was no difference in the number of extravasations between groups (Fig. 4h, i).

### The Effect of Neonatal HI and EPC Administration on BBB Related Gene Expression

**Fig. 5:**
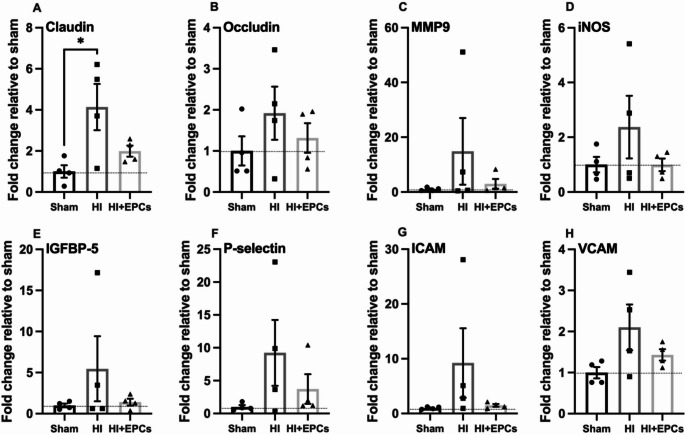
Exposure to HI increased expression of endothelial tight junctions. Choroid plexuses were collected 7 days post-HI to measure mRNA expression of key blood brain barrier related genes using qPCR including Claudin (**A**), Occludin (**B**), MMP9 (**C**), iNOS (**D**), IGFBP-5 (**E**), P-selectin (**F**), ICAM (**G**) and VCAM (**H**). *n* = 4/group. *P < 0.05. All data are presented as mean ± SEM. Data were analysed using a one-way ANOVA with Tukey’s post-hoc analysis

Beyond the BBB, there is another important brain barrier, the brain/CSF barrier, that is maintained via the choroid plexus. The effect of neonatal HI brain injury on the choroid plexus is understudied and given that this is a site of significant immune cell infiltration [24], we wanted to understand if EPC treatment would affect the choroid plexus. It has been previously shown that bone marrow derived EPCs can incorporate and alter the choroid plexus [25]. We quantified mRNA expression of BBB related genes in the choroid plexus isolated from brains at post-mortem. We examined 16 genes and saw a significant increase in claudin expression in the HI group compared to sham (*P* = 0.026; Fig. 5) and this increase was no longer significant with EPC treatment compared to the sham group. There was no significant change between groups in mRNA expression of any of the other measured genes (Fig. 5, supplementary Fig. 2).

## Discussion

Neonatal HI causes various adverse neuropathologies, including damage to the BBB and, more widely to the cells of the NVU. Previous studies have demonstrated that UCB derived EPCs reduce HI brain injury; however the effect of EPC administration on the BBB and NVU has not been investigated in the setting of HI. We hypothesised that administering EPCs 24 h after inducing HI in neonatal rat pups would repair damage to the BBB and more widely to the cellular components of the NVU. In this study we demonstrated that EPCs were able to mitigate HI-mediated damage to the brain, by reducing ipsilateral tissue loss, microglial activation and astrogliosis. However, HI resulted in an unexpected upregulation of BBB markers, which were, in turn, downregulated by EPC administration.

In this study we showed a significant injury profile in the HI group, as seen by increased tissue loss and microglia activation. This was significantly reduced following administration of EPCs and normalised to sham levels even when EPCs were administered 24 h after the initiation of injury. These results are consistent with our previous study that showed that EPCs can reduce tissue loss and neuroinflammation [15]. Further, in this study we examined the activation state of microglia and demonstrated at 7 days post-HI, microglia displayed a predominantly activated phenotype, with minimal presence of resting or intermediate microglia. This is consistent with a previous study where we demonstrated a progressive shift in microglia morphology following neonatal HI, with the peak in resting microglia at 6 h post-HI, the peak in intermediate microglia at 12 h post-HI, and the peak in activated microglia at 72 h post-HI [21]. In comparison, EPC administration significantly suppressed microglial activation and as such, sham and EPC treated animals displayed predominantly resting phenotypes.

Acosta et al. reported similar results in their study investigating EPC therapy for adult stroke in rats. They showed that EPC administration decreased M1 pro-inflammatory microglia, as seen by a decrease in major histocompatibility complex (MHC) class II + staining in the cortex and the striatum 7 days after stroke [26].

In contrast, a study by Ma et al. found that 14 days post-ischemia in a model of neonatal stroke, EPC administration resulted in a significant increase in total microglia compared to both sham and vehicle treated animals [27]. However, further analysis of these microglia demonstrated an M2 anti-inflammatory profile. This delayed increase in anti-inflammatory microglia, which did not emerge until 14 days post-stroke, may be due to EPCs increasing recruitment of anti-inflammatory microglia to the site of injury.

Astrocytes are a key cell of the NVU and play a role in BBB formation and maintenance. They have important transport functions including the facilitation of glucose delivery to neurons [28]. In addition to this, astrocytes become reactive when exposed to adverse environments and can exert an inflammatory response [29, 30]. We demonstrated a significant increase in astrocyte activation in the HI group compared to sham, with astrocytes in the HI group displaying a reactive phenotype. This is consistent with other studies that have shown that neonatal HI results in astrogliosis [31–33]. Importantly, we demonstrated that EPC administration significantly reduced astrocyte activation. The ability of EPCs to reduce astrogliosis and microglial activation highlights the anti-inflammatory and immunomodulatory properties of EPCs. These properties have been well documented while EPCs secrete various factors, such as VEGF, IL-10, IL-6 and TGF-β, that are known to be anti-inflammatory and immunosuppressive, as well as being pro-angiogenic [34].

In addition, we investigated the expression of AQP4 which is a water channel protein found on astrocytic end feet in the brain. AQP4 plays a critical role in regulating cerebral oedema, a pathology that is associated with ischemia. Previous studies have shown that AQP4 expression and astrocyte swelling increase following neonatal HI [35]. In the current study, we showed that AQP4 expression was upregulated following HI, indicating increased oedema in the brains of these animals, but we did not directly measure this outcome. However, we were able to show a positive correlation between the presence of AQP4 and GFAP-positive cells within the somatosensory cortex. This could indicate oedema in the astrocytes in the HI group, however we did not directly measure this. We subsequently showed a decrease in AQP4 expression in the EPC treated animals. It is known that exposure to hypoxia results in subcellular re-localisation of AQP4 in astrocytes, thus increasing membrane permeability and subsequent oedema [36]. In addition, upregulation of the pro-inflammatory factors IL-1β and TNFα are associated with upregulation of AQP4 [37]. As previously mentioned, EPCs are known to be anti-inflammatory [38], therefore EPCs may modulate AQP4 expression through inflammatory pathways.

We have previously characterised the progression of injury to the BBB over the first 72 h post-HI in a neonatal rat model [21]. As injury progressed, we observed an increase in vessel leakiness as seen by the presence of microbleeds and albumin extravasation with a peak at 12 h post-HI. This peak permeability was also shown by Ek et al. where BBB permeability was assessed in the hours (2, 6, and 24 h) and days (3 and 7 days) post-HI [39]. Peak vessel permeability was seen at 6–24 h, however by 3 and 7 days post HI it was no longer different from controls [39]. In this present study we also showed no presence of vessel leakiness or change in vessel number at 7 days post-HI. This is contrary to data by Ferrari et al. who showed that BBB permeability and vessel leakiness is maintained until 7-days post-HI, but had resolved by 21 days post-HI [6]. As there was no difference in albumin extravasation between groups, it is proposed that this is due to endogenous BBB repair rather than an EPC mediated response. Further to this we demonstrated that GLUT1 density and claudin-5 mRNA expression were upregulated in the HI group compared to sham. This was unexpected as we have previously shown that between 6 and 72 h post-HI, claudin-5 expression was downregulated, and GLUT1 expression remained unchanged [21]. EPC treated animals had significantly downregulated expression of GLUT1 compared to HI, and in this group, expression of claudin-5 was similar to sham levels. This is significant as GLUT1 is expressed exclusively on cerebral endothelial cells, therefore changes in GLUT1 expression is indicative of changes to endothelial cells [40]. It is proposed that upregulation of these BBB markers is caused by neovascularisation that is driven by endogenous repair mechanisms, and potentially endogenous EPCs, in response to HI exposure. In fact, circulating EPC levels and increased EPCs in ischaemic tissue have been closely associated with improved outcomes in ischaemic stroke patients [41]. EPCs exist in the bone marrow stem cell niche and are mobilised in response to chemokines and growth factors known to be upregulated in stroke including Stromal cell-derived factor 1 (SDF-1) and its cellular receptor C-X-C chemokine receptor type 4 (CXCR4) [42]. In models of adult stroke, SDF-1 is expressed at the site of injury for up to 30 days, allowing a wide window for EPC mobilisation [43]. However, in the setting of neonatal HI, SDF-1 expression has been shown to be upregulated 7 days after injury, but decreases to control levels by 10 days [43], suggesting the time-dependent recruitment of endogenous and exogenous EPCs may be shortened in the neonate. As such, future studies should include analysis of SDF-1 and CXCR4 in response to neonatal HI and EPC treatment. We have previously shown a decrease in laminin expression between 12 and 72 h post-HI [21], however there was no change in laminin expression between any groups in this study when assessed 7 days post-HI. Further, we did not observe changes in other markers of BBB integrity, such as occludin or Matrix metalloproteinase-9 (MMP9) and we also saw no changes in key growth factors such as brain derived neurotrophic factor (BDNF) or vascular endothelial growth factor (VEGF). Other studies have shown that 24 h post-HI there was reduced expression of the TJs claudin-5 and ZO-1 and that MMP9 expression was upregulated for 7 days, however, the peak was at 24 h [44]. Ek et al. also showed that 6 h post-HI, there was upregulation of claudin-5 gene expression in the hippocampus, as well as upregulation of occludin and ZO-1 gene expression in both the cortex and choroid plexus [39]. This suggests time-dependent changes in BBB morphology following neonatal HI, and therefore as we only conducted analyses 7 days post-HI, it is unclear what the time dependent roles of EPCs are in injury modulation and BBB repair.

There are no well-defined markers of EPCs and this is one of the main limitations in this study. Other studies have most commonly used CD133, CD34, VEGFR2 or CD31, however, these markers are also expressed in other cell types, including hematopoietic stem cells or mature endothelial cells. Some studies also characterised EPCs by their ability to form colonies; however this characteristic is also shared by various other cell types [45]. As such, there is heterogeneity in the current research on EPC therapies and future studies should attempt to better define EPCs to ensure consistency amongst research using these potential therapies. Another limitation of this study is that brains were only collected at 7 days post-HI. As discussed earlier, changes to the BBB occur in a time dependent manner, with vessel permeability peaking between 12 and 24 h post-HI. As such, the effect of HI and EPC administration should be analysed at earlier timepoints to determine key EPC administration times, and to map the progression of injury to the BBB. Early investigation of systemic cytokines will allow us to detect an upregulation of paracrine factors following EPC administration. Further, looking at systemic expression of cytokines and chemokines could indicate mobilisation of endogenous bone marrow EPCs that may be contributing to neovascularisation in response to HI injury. Another limitation of this study was that other key NVU components including neurons and pericytes were not characterised in the analyses. Future studies should include these cell types in the analyses to ensure full characterisation of the effect of HI and EPC administration on the NVU.

A future direction of this study is to consider in vitro cell expansion to increase cell numbers available for therapeutic applications. CD133 + cells are only a small fraction of UCB, comprising 0.2-1% of MNCs [46, 47], meaning that EPCs may not be a feasible therapy option without expansion. Current UCB expansion has been focused on CD34 + HSCs [48], however considering HSCs and EPCs come from a common lineage, and the significant overlap in cell surface markers, it is proposed that in vitro expansion would be achievable for EPCs. This would make EPCs a promising off the shelf therapy that could be more easily applied than whole MNCs, as it may reduce the need for matching multiple units of UCB.

## Conclusion

Overall, we demonstrate that administration of UCB derived EPCs 24 h after neonatal HI was able to ameliorate HI mediated upregulation of BBB markers that we observed 7 days post-HI. In addition, EPCs reduced astrogliosis and upregulation of the water channel protein AQP4, as well as reducing gross neuropathology and neuroinflammation. This study demonstrated for the first time that UCB derived EPCs show promise as a therapy that can protect the BBB and NVU in the setting of neonatal HI, however further research is required to understand the time-dependent changes to the BBB after HI, and how EPCs modulate these changes.

## Electronic Supplementary Material

Below is the link to the electronic supplementary material.

Supplementary Material 1

## Data Availability

The datasets generated for this study are available on request to the corresponding author.
