# Supplementary material for: Umbilical Cord Blood Endothelial Progenitor Cells Modulate Neurovascular Unit Damage in a Neonatal Rat Model of Hypoxia Ischemia
Source: Stem Cell Rev Rep. 2025 Jul 31;21(7):2310–21. doi: 10.1007/s12015-025-10939-z (PMC12408744; doi:10.1007/s12015-025-10939-z)
Supplement: Supplementary file 1 — Supplementary Material 1 [file 12015_2025_10939_MOESM1_ESM.docx]

| Table S1. Primer sequences for gene expression analysis | | |
| --- | --- | --- |
| Gene primer |  | Sequence |
| SELE | Forward | TGCAGGGGTACAGTGTTCAA |
|  | Reverse | TACCGTGCCAAAAACTGCTG |
| SELP | Forward | TTTTCGTCACAAAGCACCCATT |
|  | Reverse | GTGAAGAGCTTGATAGCTGGGA |
| ICAM1 | Forward | TTTGGGCTTCTCCACAGGTC |
|  | Reverse | CCACTGCTCGTCCACATAGT |
| VCAM1 | Forward | TGTGTGAAGGAGTGAATCTGGT |
|  | Reverse | CAGAACAACGGAATCCCCAAC |
| GDNF | Forward | AAGTTATGGGATGTCGTGGCT |
|  | Reverse | AGAAGCCTCTTACCGGCG |
| BDNF | Forward | AGCAGTCAAGTGCCTTTGGA |
|  | Reverse | CGCTAATACTGTCACACACGC |
| VEGFA | Forward | AGCGACAAGGCAGACTATTCA |
|  | Reverse | AATCCCAGAGCACAGACTCC |
| IGF1 | Forward | CGGGACGTACCAAAATGAGC |
|  | Reverse | CAAGCAGAGTGCCAGGTAGAA |
| IGFBP5 | Forward | CGACGCCTCTTACCTGTTCT |
|  | Reverse | TAAAACGCACCCCCACTTCT |
| PDGFA | Forward | AACGGTGAGGAACTCGACTC |
|  | Reverse | TTGTCCGCTCTGGTGAAACT |
| CLDN5 | Forward | TTGTGAGGACTTGACCGACC |
|  | Reverse | CTGTTAGCGGCAGTTTGGTG |
| OCLN | Forward | TATGCTGACCGTAGTACAGAAAGT |
|  | Reverse | TTCCACTCGGGCTCAATCC |
| PTGS2 | Forward | ATGTTCCATTTGTGAAGATTCCTGT |
|  | Reverse | TCATTTCCCTTCTCACTGGCTT |
| MMP9 | Forward | GCCTGTGGTTGGTCAGAAGA |
|  | Reverse | AGTTCAATCCCCAGATGCCC |
| ANGPT1 | Forward | AGGTGGACTGCTCTGTTTATTGA |
|  | Reverse | ATTGCCTTGAGCCCTTAGCA |
| NOS2 | Forward | GCTTGTCTCTGGGTCCTCTG |
|  | Reverse | CTCACTGGGACAGCACAGAA |
| 18S* | Forward | GTAACCCGTTGAACCCCATT |
|  | Reverse | CCATCCAATCGGTAGTAGCG |


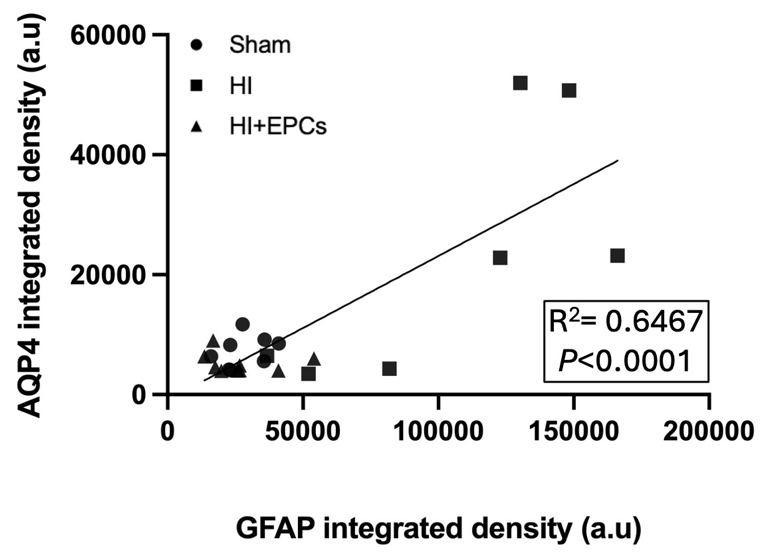


Supplementary figure 1: Correlation of AQP4 and GFAP density measured from immunofluorescence-stained slides. Density was measured using ImageJ software.


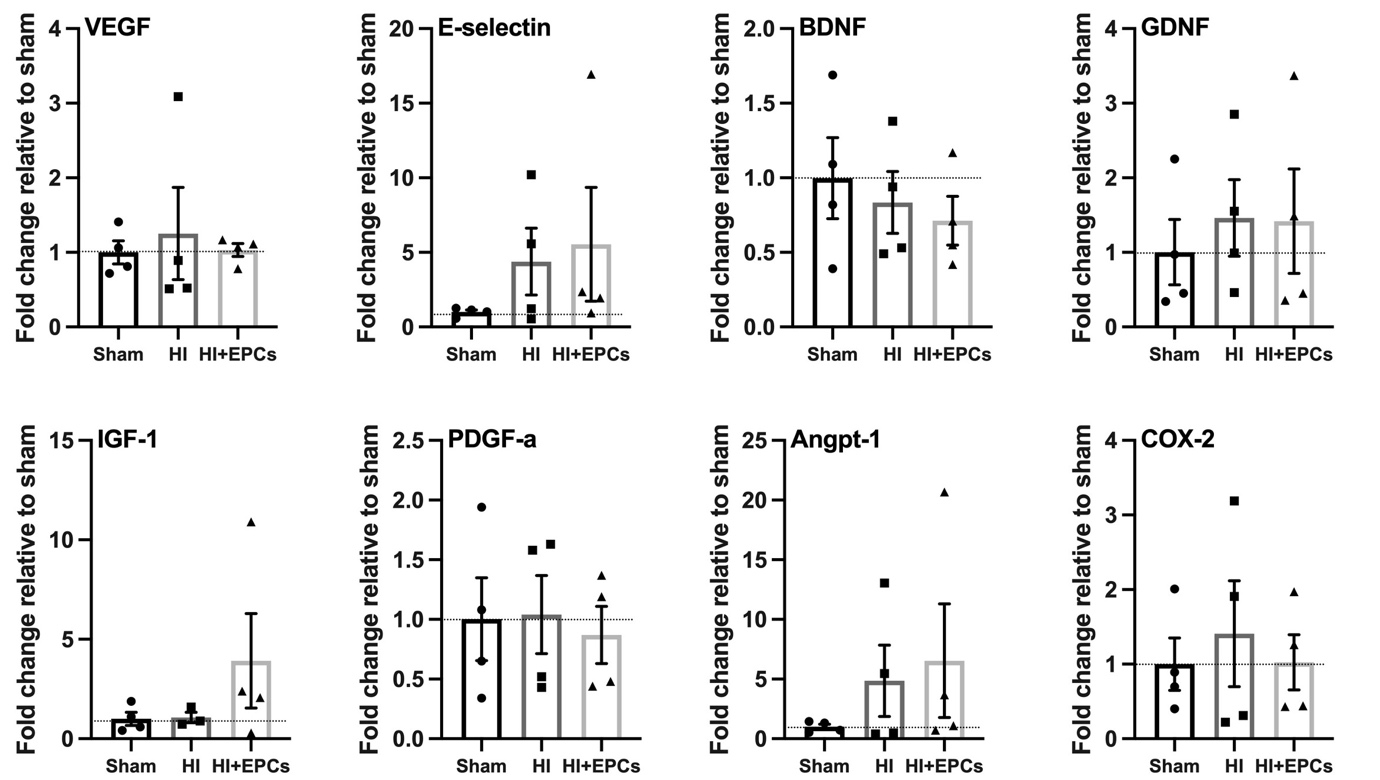


Supplementary figure 2: Additional PCR results showing expression of key angiogenic and neurogenic genes in the choroid plexus. Data is presented as a fold-change from sham expression.
